# Spectral embedding finds meaningful (relevant) structure in image and microarray data

**DOI:** 10.1186/1471-2105-7-74

**Published:** 2006-02-16

**Authors:** Brandon W Higgs, Jennifer Weller, Jeffrey L Solka

**Affiliations:** 1 School of Computational Sciences, George Mason University, Manassas, VA 20110, USA; 2 Naval Surface Warfare Center, Code B10, Dahlgren, VA 22448-5000, USA

## Abstract

**Background:**

Accurate methods for extraction of meaningful patterns in high dimensional data have become increasingly important with the recent generation of data types containing measurements across thousands of variables. Principal components analysis (PCA) is a linear dimensionality reduction (DR) method that is unsupervised in that it relies only on the data; projections are calculated in Euclidean or a similar linear space and do not use tuning parameters for optimizing the fit to the data. However, relationships within sets of nonlinear data types, such as biological networks or images, are frequently mis-rendered into a low dimensional space by linear methods. Nonlinear methods, in contrast, attempt to model important aspects of the underlying data structure, often requiring parameter(s) fitting to the data type of interest. In many cases, the optimal parameter values vary when different classification algorithms are applied on the same rendered subspace, making the results of such methods highly dependent upon the type of classifier implemented.

**Results:**

We present the results of applying the spectral method of Lafon, a nonlinear DR method based on the weighted graph Laplacian, that minimizes the requirements for such parameter optimization for two biological data types. We demonstrate that it is successful in determining implicit ordering of brain slice image data and in classifying separate species in microarray data, as compared to two conventional linear methods and three nonlinear methods (one of which is an alternative spectral method). This spectral implementation is shown to provide more meaningful information, by preserving important relationships, than the methods of DR presented for comparison.

Tuning parameter fitting is simple and is a general, rather than data type or experiment specific approach, for the two datasets analyzed here. Tuning parameter optimization is minimized in the DR step to each subsequent classification method, enabling the possibility of valid cross-experiment comparisons.

**Conclusion:**

Results from the spectral method presented here exhibit the desirable properties of preserving meaningful nonlinear relationships in lower dimensional space and requiring minimal parameter fitting, providing a useful algorithm for purposes of visualization and classification across diverse datasets, a common challenge in systems biology.

## Background

Dimensionality reduction (DR) is the process of rendering high dimensional complex data in a low dimensional space. Provided the process is calculated accurately, this low dimensional representation is preferred for use in inference and summarization for multiple reasons, among which are ease of visualization in a reduced variable space and clarity (i.e. ready interpretation) of clustering or classification. Other benefits include the insights into the data structure that can be obtained from the projected axes and the obvious denoising effect attained in some types of DR. Reduction strategies often rely on linear approaches defined by a method that represents *x*_1_, ..., *x*_*n *_∈ ℝ^*q *^as x^1,…,x^n
 MathType@MTEF@5@5@+=feaafiart1ev1aaatCvAUfKttLearuWrP9MDH5MBPbIqV92AaeXatLxBI9gBaebbnrfifHhDYfgasaacH8akY=wiFfYdH8Gipec8Eeeu0xXdbba9frFj0=OqFfea0dXdd9vqai=hGuQ8kuc9pgc9s8qqaq=dirpe0xb9q8qiLsFr0=vr0=vr0dc8meaabaqaciaacaGaaeqabaqabeGadaaakeaacuWG4baEgaqcamaaBaaaleaacqaIXaqmaeqaaOGaeiilaWIaeSOjGSKaeiilaWIafmiEaGNbaKaadaWgaaWcbaGaemOBa4gabeaaaaa@3557@ in such a way that each x^i
 MathType@MTEF@5@5@+=feaafiart1ev1aaatCvAUfKttLearuWrP9MDH5MBPbIqV92AaeXatLxBI9gBaebbnrfifHhDYfgasaacH8akY=wiFfYdH8Gipec8Eeeu0xXdbba9frFj0=OqFfea0dXdd9vqai=hGuQ8kuc9pgc9s8qqaq=dirpe0xb9q8qiLsFr0=vr0=vr0dc8meaabaqaciaacaGaaeqabaqabeGadaaakeaacuWG4baEgaqcamaaBaaaleaacqWGPbqAaeqaaaaa@2FBC@ is obtained by projecting *x*_*i *_into a common linear subspace of ℝ^*q*^. Commonly used methods on data types relevant to bioinformatics include principal components analysis (PCA) [1] and classical multidimensional scaling (MDS) [2], which calculate linear projections of the data; clearly such projections are unsuitable for nonlinear or curved surfaces.

These methods generally are based on minimization of a global cost function, wherein large distances can drive the embedding, minimizing the effect of local distance structures [3-5]. Where local data structures are not best summarized linearly (yet important to the interpretation of the experimental results), nonlinear methods that are kernel-based (e.g. kernel PCA) [6] and graph theoretic like spectral embedding [3-17] can be more appropriate. These methods attempt to model the underlying manifold by fitting a kernel parameter to optimize performance (e.g. as assessed by some performance accuracy metric) [6]. Unfortunately it is usually necessary to re-fit one or more tuning parameter(s) to each different data type or experiment set, making it difficult to propose a more generalized method across multiple data types. It is also difficult to avoid over-fitting the model to the data in this scenario. In addition, when attempting to determine class structure in the low dimensional space calculated from these nonlinear approaches, different classifiers may require separate spatial representations in order to appropriately partition the classes (e.g. quadratic discriminant analysis (QDA) compared to linear discriminant analysis (LDA)). Such parameter(s) modifications are optimized with a specific range of values that can be different for each classifier.

Two examples of high dimensional data types that fall into this nonlinear domain include DNA microarrays and image data. Microarrays contain the simultaneous measurement for thousands of mRNA transcripts [18-20], which can be viewed as *n *arrays with *q *dimensions (where *n*<<*q*). Many of the biological processes (feedback loops, oscillators, and repressilators) represented by measurements generated with microarrays are nonlinear, providing a great challenge in expressing associations between biological entities in a linear domain. Nilsson *et al*. demonstrated the importance of this concept in their comparison of MDS with a nonlinear algorithm, isometric feature mapping (ISOMAP) [21,22]. ISOMAP uses nonlinear distances as estimated in the ambient space along with a linear MDS to a Euclidean target projection space [22]. This nonlinear method was shown to render more robust partitioning of disease class structure on the low dimensional manifold, when class membership predictions were evaluated against those obtained from linear projections from MDS.

Images are another data type that can be represented in *q *dimensions as well, where each image *n *is a vector. This data type can exhibit comparable complexities to the microarray example, particularly when imaging tissues and organs. The Euclidean distance between two similar images is seldom the optimal comparison criterion. Simple variations on the main image features, such as those caused by registration issues (rotation and shifting), can alter the pixel alignment, thereby modifying the definition of distance between the original image and the rotated one, and distorting the apparent relationship. The ideal method for DR should be capable of extracting meaningful patterns in multiple data types (such as these mentioned), should not be confined to a linear domain, and should exhibit tuning parameter-fitting independence to minimize parameter optimization specific to each example and classification method.

Given this goal, we examined the performance of a spectral method presented by Lafon [3,4] and have shown that it is successful in extracting meaningful structure in these two disparate data types, both having high dimensionality paired with low replication, with a method for calculating the tuning parameter that does not have to be varied across classifiers to achieve correct results. Previous work by Lafon has demonstrated how ordered structure from both helix and trefoils curves in ℝ^3 ^can be accurately preserved in the embedded space (ℝ^2^) with a spectral method [3]. We extend this work to address biological examples of higher dimensionality, where accuracy in embedded results is evaluated using a known ordering and classification structure. In a more global sense, we demonstrate that the spectral method is able to preserve the implicit ordering within biological image data and can accurately classify different taxonomic species within microarray data. These results are compared to two linear approaches (PCA with either correlation or covariance distance metrics), one nonlinear counterpart to classical MDS (i.e. nonmetric MDS), and two similar nonlinear approaches (kernel PCA with a Gaussian radial basis function kernel; weighted graph Laplacian as presented by Ng *et al*. [11]), for the latter two of which numerous variations are often promoted in the mathematical/statistical literature for their successful application to a number of nonlinear data types [11-13,15-17]. We demonstrate that for our datasets, the spectral approach presented here is not dependent upon tuning parameter(s) optimization to allow success across any of three separate classifiers chosen. This is a considerable advantage to an investigator who needs to make cross-experiment or multi-data type comparisons that benefit from a tuning parameter-independent nonlinear DR approach.

## Results and discussion

### Image dataset

The image dataset was used to test the ability of each of the projection methods to predict the correct image ordering, based on the size increment of the brain. Since the largest source of variability separating each image in the series is the increase in feature surface area, as a result of the head size, only the first eigenfunction for each method was used in the comparison. This calculation reduces the dimensionality from ℝ^16,384 ^to ℝ^1^. To assess the accuracy of each method, a non-parametric measure of association (Spearman's rho coefficient) was used, by which the scores from the primary eigenfunction were ranked and correlated against the correct ordering. A straight-line fit is indicative of perfect image ordering.

**Figure 1:**
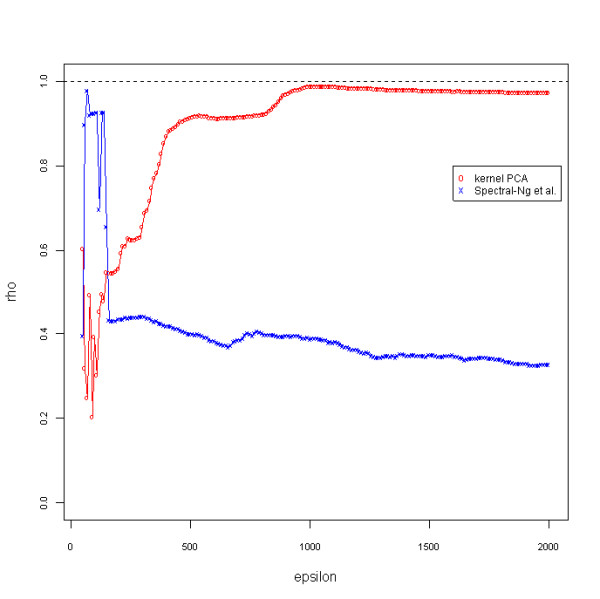
**Parameter optimization plot for image example**. Regression coefficients for image ordering determined by different epsilon values for kernel PCA and the spectral method from Ng *et al*. Epsilon values were increased to 300,000 to assess image ordering accuracy (data not shown), but truncated for the plot to better visualize the global maxima. The dashed black horizontal line indicates a rho statistic value of 1, though neither method reaches this threshold. Large fluctuations in the rho statistic are observed for both methods at minimal values of epsilon. For kernel PCA a non-optimal solution is determined in the variable region, while for the spectral method from Ng *et al*., a maximum is determined in this region. The variability in the rho values at these minimal values can be associated with the optimal convergence of remote and local distances in the weight matrices (Figure 6) of kernel PCA and the spectral method from Ng *et al *(*L*). Small values of the epsilon parameter provide minimal convergence of the *L *matrix distribution tails (very small distances and very large distance), which is optimal for the spectral method from Ng *et al *for this example. For kernel PCA, larger values of the epsilon parameter provide convergence of large distances and greater convergence of small distances in the Gaussian radial basis function kernel matrix, which is determined to be optimal for this example.

Both the kernel PCA and the spectral method from Ng *et al*. require fitting for the smoothing epsilon term to optimize performance for the dataset. The results for this parameter optimization are provided in Figure 1. The maximum rho coefficient possible indicates the appropriate value for this epsilon term for each method. Based upon the observation that neither line reaches a maximum value of 1, it is apparent that neither method is capable of determining the correct ordering of all of the images.

The remaining methods: PCA-correlation, PCA-covariance, nonmetric MDS, and the spectral method from Lafon [3,4] do not require parameter fitting that is dependent upon performance (as is necessary for the previous two nonlinear methods), so the images can be directly rendered into a low dimensional representation.

**Figure 2:**
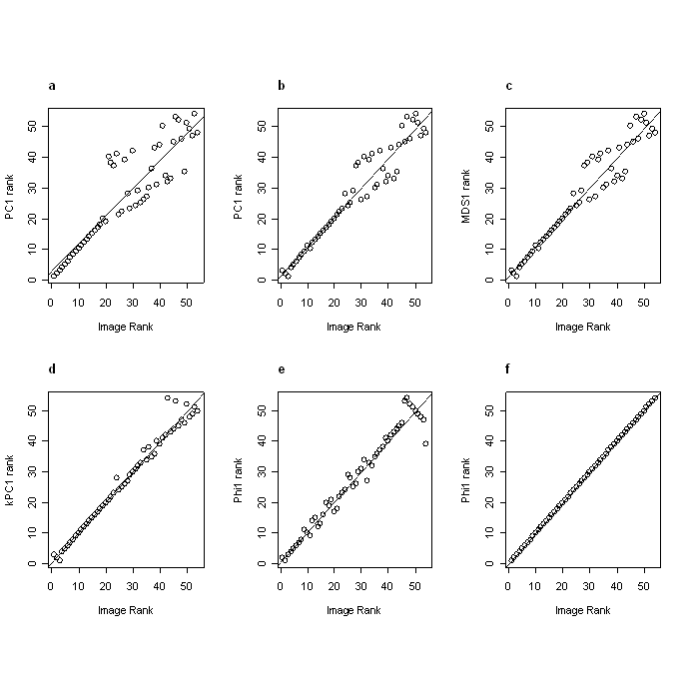
**Scatter plots of image ordering for six projection methods**. **(a) **Regression of ranked eigenfunction values calculated by PCA-correlation vs. actual ordering. **(b) **Regression of ranked eigenfunction values calculated by PCA-covariance vs. actual ordering. Note that the scores have been reverse sorted for consistency with the other plots. **(c) **Regression of ranked eigenfunction values calculated by nonmetric MDS vs. actual ordering. **(d) **Regression of ranked eigenfunction values calculated by kernel PCA (*ε *= 1,040) vs. actual ordering. **(e) **Regression of ranked eigenfunction values calculated by the spectral method from Ng *et al*. (*ε *= 70) vs. actual ordering. **(f) **Regression of ranked eigenfunction values calculated by the spectral method from Lafon [3,4] vs. actual ordering.

**Figure 3:**
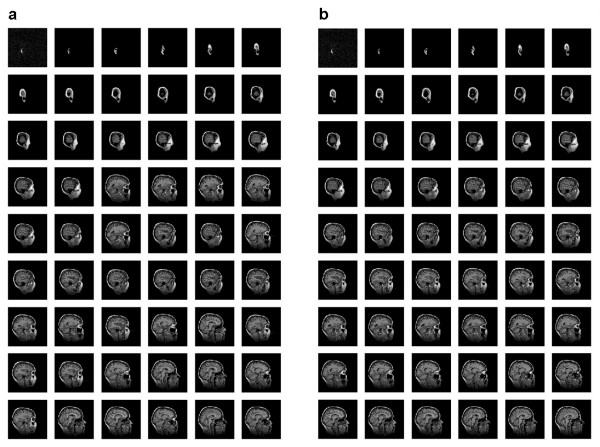
**Images ordered by the primary eigenfunction for PCA-correlation and the spectral method from Lafon [3,4]**. **(a) **The ordering is not correctly preserved with PCA-correlation (in this series, moving from left to right and top to bottom). **(b) **The order is correctly preserved with the spectral method from Lafon [3,4] (in this series, moving from left to right and top to bottom).

Table 1 and Figure 2 illustrate the results for all of the projection methods. PCA-correlation has the lowest coefficient (rho = 0.902). The plot (Figure 2a) demonstrates that the global size change is minimal after image 20 and the subtler differences in shape are not picked up by this method. This method thus fails to predict the correct progression between the images after this point. Both PCA-covariance and nonmetric MDS (Figures 2b and 2c, respectively) show exactly the same ability to solve for the correct ordering of images (rho = 0.966). Since these two methods give the same results, it is implied that the ordering of dissimilarity values (as fit with classical MDS) and the *rank *of the ordering of dissimilarity values (as fit with nonmetric MDS) are identical, which implies that there is no benefit in using nonmetric MDS to recover the image ordering. These two methods fail to retain the ordering after image 22, although the deviations at this point are not as drastic as those observed for the PCA-correlation results. The two other nonlinear methods (Figures 2d and 2e), each of which requires parameter optimization, predict the image ordering more correctly than do the two linear methods, indicating that this dataset is not best summarized with linear methods. Where there are meaningful local relationships, or nonlinearities, that the linear methods fail to preserve in a low dimensional mapping, nonlinear methods will be a more appropriate analysis choice. However, neither kernel PCA nor the spectral method from Ng *et al*. accurately preserves the correct image ordering over the entire series (rho = 0.989 and rho = 0.980, respectively). In this study, only the spectral method from Lafon was able to correctly solve the implicit ordering of the complete set of images (Figure 2f). This spectral method shares the properties of nonlinearity with kernel PCA and the spectral method from Ng *et al*. (which is initially anchored on the transformation of the Euclidean distance to some form of Gaussian kernel), however, it does not require parameter fitting of the epsilon term in order to produce optimal performance. Instead, the minimum non-zero squared distance is calculated for the smoothing term (for this example, *ε *= min_*i*≠*j *_||*x*_*i *_- *x*_*j*_||^2 ^= 140,245), meaning that this method is dependent only upon the distribution of squared Euclidean distances. The image ordering as produced by both PCA-correlation and the spectral method from Lafon is provided in Figure 3.

**Table 1:** Spearman rho values used for evaluation of CATSCAN image ordering

**Method**	**rho**
PCA (cor)	0.902
PCA (cov)	0.966
Nonmetric MDS	0.966
Kernel PCA (*ε *= 1,040)	0.989
Spectral-Ng *et al*. (*ε *= 70)	0.980
Spectral-Lafon	1.000

Two linear methods and four nonlinear methods are considered. For both kernel PCA and the spectral method from Ng *et al*., the methods were optimized at the epsilon (ε) values provided.

### Microarray dataset

The microarray dataset was used to evaluate the ability of the projection methods to accurately classify samples derived from three taxonomically separate species into their respective groups, without conducting any preliminary feature selection (a procedure usually conducted in order to better partition the groups). In each case, the results of a method were calculated such that dimensionality was reduced from ℝ^12,625 ^to ℝ^2^. Thereafter three classifiers using leave-one-out cross-validation (LOO-CV) were calculated on this projected space, both to assess the classification accuracy for each method and to compare the differences in value of the optimal parameters for kernel PCA and the spectral method from Ng *et al*. A nonlinear classifier, *k*-nearest neighbors (KNN), was calculated, setting *k *= 2 and *k *= 3; two settings of *k *were used since each DR method renders the groupings differently, thereby favouring two nearest neighbors for some methods and three nearest neighbors for others. The average error rates were computed across 1,000 trials, to account for the variation arising from ties broken at random in the assignment of the nearest winning class which results in slightly different classification results for subsequent trials. In the event that the mean error rate is calculated to be greater than zero, even when one or more of the trials provide a smaller error rate, a range is denoted in the results (e.g. 0%–4.66%) to indicate that the occurrence of a lower error rate is possible. Another commonly utilized classifier, QDA, was calculated, as well as the linear classifier counterpart, LDA, to allow comparison of the dependence of the results of different classification methods on the tuning parameter fitting in the first DR step.

**Figure 4:**
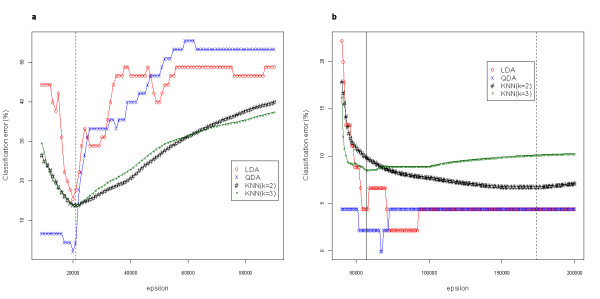
**Parameter optimization plot for microarray example**. Classification error rates for primary fibroblast cell lines between three separate species determined by different epsilon values for **(a) **the spectral method from Ng *et al*. and **(b) **kernel PCA. Epsilon values were increased to 5 × 10^9 ^to assess classification accuracy (data not shown), but truncated for the plot to better visualize the global minima for each classifier method. The spectral method from Lafon has a 0% error rate across all methods except LDA, where only one sample is misclassified.

As was done with the image data, optimal parameters were determined for kernel PCA and the spectral method from Ng *et al*. These values were evaluated with each classification algorithm separately. In determining the most appropriate epsilon value for optimal classification accuracy, the optimal value for the spectral method from Ng *et al*. varies according to the classifier used (Figure 4a). There is a small window at a value of *ε *~20,000 in which both QDA (blue line) and LDA (red line) reach respective minimum total classification error rates of 2.22% and 15.56%. However, the KNN classifiers for *k *= 2 (black line) and *k *= 3 (green line) require slightly higher epsilon values (21,000 indicated by a dashed vertical line) to reach their respective minimum total classification error rates (2.22%–13.93% and 8.88%–13.79%, respectively). It can be argued that increasing the trials of the KNN classifier might better adjust this minimum point in the two KNN curves, where it coincides with the QDA and LDA window for minimum error. However, without calculating error rates with three separate classifiers, but rather independently determining the value for a given classifier, this window would be unknown, in which case different optimal parameter values for each classifier would be suggested. For example, using LDA as a classifier to determine the minimum error rate, a value of *ε *= 20,000 can be chosen as the optimal parameter for the spectral method from Ng *et al*.; however, for a KNN (*k *= 3) classifier in the same example, this parameter value would not fall within the range of the minimum error rate. Instead, a value of *ε *= 20,000 would provide a local minimum error of 14.08%, as compared to the global minimum error of 13.79%.

The parameter optimization for kernel PCA shows similar trends to the optimal values from the spectral method from Ng *et al*. However, the differences in optimal parameter solutions between the KNN classifier and both QDA and LDA is much more pronounced with this DR method. For the QDA classifier, the epsilon value is optimized to provide a minimal error rate (0%) in the window of *ε *= 67,000 – 68,000, while for the LDA classifier, the epsilon value is optimized to provide a minimal error rate (2.22%) in the window of *ε *= 73,000 – 92,000. In addition, the epsilon values that provide the minimal KNN error rates for *k *= 2 (2.22%–6.79%) and *k *= 3 (8.56%) are at *ε *= 174,000 (indicated by a dashed vertical line in Figure 4) and *ε *= 57,000 (indicated by a solid vertical line in Figure 4), respectively. This result from kernel PCA is consistent with those obtained from the spectral methods of Ng *et al*., and demonstrates that each of these two nonlinear approaches have a dependence between the outcome of the classification algorithm and an appropriately optimized parameter. In addition, when comparing training set classification results to LOO-CV, the optimal parameter values are drastically different for both kernel PCA and the spectral method of Ng *et al*. More importantly, the parameter selection is completely dataset-dependent. Note that here the scale is vastly different from that seen in the image example, thus the optimal parameter is as well. For these examples we examined two very disparate data types, but the same conclusion of dataset-dependence would almost certainly occur if two microarray datasets were compared, since there would still be differences in distance distributions (the possibility of two microarray datasets having identical distributions of Euclidean distances is highly unlikely). In other work we have examined additional microarray datasets and examined functional sub categories as well as disease state, cited here for those who are interested [5].

**Figure 5:**
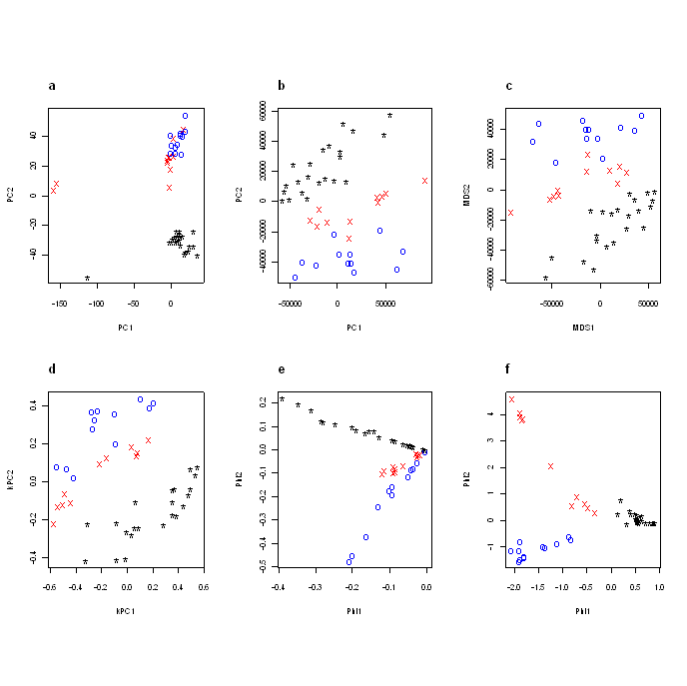
**Two-dimensional projections of the Affymetrix primary fibroblast cell lines, using six DR methods**. For each plot, red 'x' symbols denote samples from bonobo (*Pan paniscus*), blue 'o' symbols denote samples from gorilla (*Gorilla gorilla*), and black '*' symbols denote samples from human (*Homo sapien*). **(a) **Two-dimensional projection calculated with PCA-correlation. **(b) **Two-dimensional projection calculated with PCA-covariance. **(c) **Two-dimensional projection calculated with nonmetric MDS. **(d) **Two-dimensional projection calculated with kernel PCA. Epsilon parameter was selected at optimal classification using QDA (*ε *= 67,000). **(e) **Two-dimensional projection calculated with the spectral method from Ng et al. Epsilon parameter was selected at optimal classification using QDA (*ε *= 20,000). **(f) **Two-dimensional projection calculated with the spectral method from Lafon.

All individual classification results are summarized in Table 2, and the two-dimensional projections for each method are shown (Figure 5). For the kernel PCA and spectral method from Ng *et al*., the projection plots were generated with the epsilon terms optimized for QDA (*ε *= 67,000 for kernel PCA and *ε *= 20,000 for the spectral method from Ng *et al*). From the total error rate results reported in Table 2, it is apparent that the nonlinear DR methods of kernel PCA and the spectral method from Lafon perform more accurately than do the two linear methods (and nonmetric MDS) across all three of the classification algorithms (though using KNN with *k *= 2, PCA-covariance and nonmetric MDS can achieve a minimal error rate of 2.22%, similar to kernel PCA). Of these three nonlinear DR methods tested (not including nonmetric MDS), the spectral method from Lafon [3,4] outperforms both kernel PCA and the spectral method from Ng *et al*. Not only does the Lafon spectral method project the different species into well-partitioned groups (Figure 5f) for a 0% error rate across all classification algorithms (excepting a single misclassified sample with the LDA classifier), but the KNN classifier does not exhibit any deviation in classification results across 1,000 iterations, unlike the results obtained with any of the other methods. These results, in addition to the property that tuning parameter optimization is only dependent on the distribution of squared Euclidean distances (for this example *ε *= min_*i*≠*j *_||*x*_*i *_- *x*_*j*_||^2 ^= 1.29 × 10^9^, indicates that the spectral method from Lafon has significant advantages in tuning parameter fitting as a nonlinear DR method by our two criteria.

**Table 2:** LOO-CV classification error rates for six DR methods and three classifiers (fibroblast data)

		**PCA (cor)**	**PCA (cov)**	**Nonmetric MDS**	**kernel PCA**^†^	**Spectral Ng *et al*.**^‡^	**Spectral Lafon**
*KNN (*k *= 2)	*Pan paniscus*	27.27%–41.02%	0%–13.71%	0%–13.69%	0%–9.13%	0%–9.02%	0%
	*Gorilla gorilla*	8.33%–29.39%	8.33%–12.61%	8.33%–12.42%	8.33%–12.49%	8.33%–29.33%	0%
	*Homo sapiens*	4.55%	0%–2.28%	0%–2.22%	0%–2.26%	0%–4.66%	0%
	Total	20.09%	2.22%–7.83%	2.22%–7.74%	2.22%–6.79%	2.22%–13.93%	0%
*KNN (*k *= 3)	*Pan paniscus*	27.27%	18.18%	18.18%	9.09%	9.09%	0%
	*Gorilla gorilla*	8.33%	16.67%	16.67%	16.67%	25%	0%
	*Homo sapiens*	4.55%	4.55%	4.55%	0%	0%–3.04%	0%
	Total	11.11%	11.11%	11.11%	8.56%	8.88%–13.79%	0%
**QDA**	*Pan paniscus*	72.73%	9.09%	9.09%	0%	0%	0%
	*Gorilla gorilla*	16.67%	8.33%	8.33%	0%	8.33%	0%
	*Homo sapiens*	4.55%	0%	0%	0%	0%	0%
	Total	24.44%	4.44%	4.44%	0%	2.22%	0%
**LDA**	*Pan paniscus*	18.18%	9.09%	9.09%	0%	0%	**9.09%
	*Gorilla gorilla*	25%	8.33%	8.33%	8.33%	41.67%	0%
	*Homo sapiens*	0%	0%	0%	0%	9.09%	0%
	Total	11.11%	4.44%	4.44%	2.22%	15.56%	**2.22%

*The *k*-nearest neighbors (KNN) classifier was calculated over 1,000 independent trials for the mean error rates to be determined. Range of error values indicate that one or more trials provided a smaller error rate, though the average was computed.

† KNN *k *= 2: *ε *= 174,000; KNN *k *= 3: *ε *= 57,000; QDA: *ε *= 67,000; LDA: *ε *= 73,000

^‡ ^KNN *k *= 2: *ε *= 21,000; KNN *k *= 3: *ε *= 21,000; QDA: *ε *= 20,000; LDA: *ε *= 20,000

**The 9.09% error rate corresponds to a single *Pan paniscus *sample that is misclassified by LOO-CV using LDA as a classifier.

## Conclusion

Within these examples, the spectral method from Lafon is demonstrated to extract more meaningful structure, relative to two linear and three nonlinear methods, for calculating low dimensional representations of high dimensional data types, such as image and microarray data, for purposes of determining ordered patterns or classification. As a nonlinear method it is shown to be a reasonable choice for biological and image data types, where it is important to preserve nonlinear relationships and local geometries in a low dimensional embedding. Though the nonlinear methods of kernel PCA and Ng's spectral method also may be well suited for these data types, they suffer the primary drawback of requiring dataset- and classifier-specific tuning parameter optimization, making the validity of cross-experiment comparisons problematic. Other nonlinear manifold methods, such as ISOMAP [22] and Local Linear Embedding (LLE) [23], have similar optimization requirements as drawbacks, although tuning parameter optimization and classification accuracy for these two methods was not assessed here. This data fitting step can be not only time consuming, but also, as we have shown, varies according to which classification algorithm is used as well as which dataset is examined. In this work, the spectral method from Lafon is shown to outperform competing methods and exhibit independence to tuning parameter fitting across three separate classifiers and two unrelated high dimensional data types. Much like any method of DR, this method is not proposed to always elucidate the most meaningful structure across all high dimensional data types. Methods such as boosting and bagging [24] and the relative distance plane (RDP) [25] may be better suited for certain high dimensional datasets. Rather, the results presented here demonstrate success in two disparate datasets of high dimensionality and the authors' hope is that this presentation will encourage others to extend applications of this method in research within the computational biology community.

## Methods

### Data types

The image data was obtained from the Computer Vision Laboratory at the University of Massachusetts at Amherst [26]. A total of 54 slice-by-slice CATSCAN images were obtained for the human head, where each image has dimensions of 128 × 128 pixels. The average for each image was calculated and the median of these averages was determined to be 11.82. Each image was then scaled to a target mean of this value.

The microarray data are from genomic primary fibroblast cell lines [27,28] and were generated with Affymetrix oligonucleotide hgu95v2 arrays for 46 samples: 23 human (*Homo sapien*), 11 bonobo (*Pan paniscus*), and 12 gorilla (*Gorilla gorilla*) donors. This is a publicly available dataset within the 'fibroEset' package in R [29]. It should be noted that two identical human donor arrays are in this dataset, so one was removed, reducing the dataset to 45 total samples.

The data was provided in R already normalized by the Affymetrix GeneChip MAS 5.0 algorithm. "Normalization was done by calculating multiplicative scaling factors on the basis of the median intensity of the 60th to 95th percentile of gene-expression scores" and intensities were floored to 100 fluorescent units [29]. No further filtering or scaling was conducted on this dataset, which consists of 12,625 expression points for each of 45 arrays.

### Spectral methods

The spectral implementation of the weighted graph Laplacian from Lafon [3,4] is calculated as follows: Given a set of points X = {*x*_1_, *x*_2_, ..., *x*_*n*_} ∈ ℝ^*q*^, let *G *= (*E*, *V*) be a graph with edge weights or lines *E*, between pairs of vertices *V*. Consistent with standard terminology from graph theory, we can construct a graph, where each pair of vertices (*x*_*i*_, *x*_*j *_∈ *V*(*G*)) is assigned a weight specific to the distance between them [30]. The matrix, *K*_1 _is calculated from these edge weights by a Gaussian kernel estimate

K1=K1(G)=(e−(‖xi−xj‖2/ε))
 MathType@MTEF@5@5@+=feaafiart1ev1aaatCvAUfKttLearuWrP9MDH5MBPbIqV92AaeXatLxBI9gBaebbnrfifHhDYfgasaacH8akY=wiFfYdH8Gipec8Eeeu0xXdbba9frFj0=OqFfea0dXdd9vqai=hGuQ8kuc9pgc9s8qqaq=dirpe0xb9q8qiLsFr0=vr0=vr0dc8meaabaqaciaacaGaaeqabaqabeGadaaakeaacqWGlbWsdaWgaaWcbaGaeGymaedabeaakiabg2da9iabdUealnaaBaaaleaacqaIXaqmaeqaaOGaeiikaGIaem4raCKaeiykaKIaeyypa0JaeiikaGIaemyzau2aaWbaaSqabeaacqGHsislcqGGOaakdaqbdaqaaiabdIha4naaBaaameaacqWGPbqAaeqaaSGaeyOeI0IaemiEaG3aaSbaaWqaaiabdQgaQbqabaaaliaawMa7caGLkWoadaahaaadbeqaaiabikdaYaaaliabc+caVGGaciab=v7aLjabcMcaPaaakiabcMcaPaaa@49D3@ if *i *≠ *j*, where *K*_1*ii *_= 0 and *ε *= min_*i*≠*j *_||*x*_*i *_- *x*_*j*_||^2 ^> 0

The epsilon term is chosen at the minimum squared Euclidean distance, as opposed to the average minimum distance (specified by Lafon) to induce maximum convergence of distance (see section on **Comparison of transformed distances **for an example). The vector, *v *is calculated from the square root of the product between matrix components from *K*_1 _and vector components from *e*. The matrix *P *is then formed by the product of *v *and *v*_*T*_. Then, the weighted graph Laplacian matrix, *K *is calculated by component division of the matrix *K*_1 _elements

(*K*_1*ij *_where *i *= 1, ..., *q *and *j *= 1, ..., *n*) by the matrix *P *elements

(*P*_*ij *_where *i *= 1, ..., *q *and *j *= 1, ..., *n*). The calculations are given as the following:

v=K1ijei
 MathType@MTEF@5@5@+=feaafiart1ev1aaatCvAUfKttLearuWrP9MDH5MBPbIqV92AaeXatLxBI9gBaebbnrfifHhDYfgasaacH8akY=wiFfYdH8Gipec8Eeeu0xXdbba9frFj0=OqFfea0dXdd9vqai=hGuQ8kuc9pgc9s8qqaq=dirpe0xb9q8qiLsFr0=vr0=vr0dc8meaabaqaciaacaGaaeqabaqabeGadaaakeaacqWG2bGDcqGH9aqpdaGcaaqaaiabdUealnaaBaaaleaacqaIXaqmcqWGPbqAcqWGQbGAaeqaaOGaemyzau2aaSbaaSqaaiabdMgaPbqabaaabeaaaaa@370E@ where *i *= 1, ..., *q*; *j *= 1, ..., *n*; and *e *= (1, 1, ..., 1)^*T*^, then

*P *= *vv*^*T *^and

*K *= *K*_1*ij*_/*P*_*ij *_where *i *= 1, ..., *q *and *j *= 1, ..., *n*.

The *K *matrix is decomposed by singular value decomposition (*svd*)

*svd*(*K*) = *XHV*^*T *^(Note that for this symmetric positive semidefinite matrix *K*, the *svd *is the spectral decomposition, however, to remain consistent with the nomenclature specified by Lafon, the calculations with *svd *are used.)

and the *n *columns of the *X *matrix which define the left singular vectors of *K *are scaled by the first column of *X*, given by

Φj=X[,j]X[,1]
 MathType@MTEF@5@5@+=feaafiart1ev1aaatCvAUfKttLearuWrP9MDH5MBPbIqV92AaeXatLxBI9gBaebbnrfifHhDYfgasaacH8akY=wiFfYdH8Gipec8Eeeu0xXdbba9frFj0=OqFfea0dXdd9vqai=hGuQ8kuc9pgc9s8qqaq=dirpe0xb9q8qiLsFr0=vr0=vr0dc8meaabaqaciaacaGaaeqabaqabeGadaaakeaacqqHMoGrdaWgaaWcbaGaemOAaOgabeaakiabg2da9maalaaabaGaemiwaGLaei4waSLaeiilaWIaemOAaOMaeiyxa0fabaGaemiwaGLaei4waSLaeiilaWIaeGymaeJaeiyxa0faaaaa@3C4E@ where *j *= 1, ..., *n*.

This provides *n *- 1 characteristic roots of the matrix *K *given by Φ_*j*_. It should be noted that the first column of *X *is scaled by itself, creating a vector of values equal to 1. By convention, this vector is designated by Φ_0_. As such, the second column in the matrix *X *will be considered the primary Φ vector, and designated by Φ_1_. Utilizing these primary Φ vectors, the data can be embedded as points in ℝ^Φ^.

The spectral implementation of the weighted graph Laplacian from Ng *et al*. is similar up to the calculation of the *K*_1 _matrix, however, the kernel is defined by a denominator term of 2*ε*^2 ^as opposed to simply *ε *in Lafon's method. In addition, the epsilon smoothing term (*ε*) (as will be demonstrated in the **Results **section) is not optimized at *ε *= min _*i*≠*j *_||*x*_*i *_- *x*_*j*_||^2 ^> 0, as it is in our modification to Lafon's method, and thus requires fitting for each example analyzed and classifier utilized. Following the calculation of the *K*_1 _matrix, the matrix *D *is a diagonal matrix calculated from the row sums of *K*_1_,

Dii=∑j=1nK1ij
 MathType@MTEF@5@5@+=feaafiart1ev1aaatCvAUfKttLearuWrP9MDH5MBPbIqV92AaeXatLxBI9gBaebbnrfifHhDYfgasaacH8akY=wiFfYdH8Gipec8Eeeu0xXdbba9frFj0=OqFfea0dXdd9vqai=hGuQ8kuc9pgc9s8qqaq=dirpe0xb9q8qiLsFr0=vr0=vr0dc8meaabaqaciaacaGaaeqabaqabeGadaaakeaacqWGebardaWgaaWcbaGaemyAaKMaemyAaKgabeaakiabg2da9maaqahabaGaem4saS0aaSbaaSqaaiabigdaXiabdMgaPjabdQgaQbqabaaabaGaemOAaOMaeyypa0JaeGymaedabaGaemOBa4ganiabggHiLdaaaa@3D91@ where *i *= 1, ..., *q*. Then the normalized Laplacian matrix, *L *is calculated as

*L *= *D*^-1/2^*K*_1_*D*^-1/2^

The Laplacian matrix, *L *is decomposed by *svd*, and the *n *columns of the *X *matrix which define the left singular vectors of *L *have rows scaled to unit length into the matrix *Y*

*svd*(*L*) = *XHV*^*T *^(Note the point specified in the decomposition of the weighted graph Laplacian as provided by Lafon.)

Yij=Xij(∑j=1nXij2)1/2
 MathType@MTEF@5@5@+=feaafiart1ev1aaatCvAUfKttLearuWrP9MDH5MBPbIqV92AaeXatLxBI9gBaebbnrfifHhDYfgasaacH8akY=wiFfYdH8Gipec8Eeeu0xXdbba9frFj0=OqFfea0dXdd9vqai=hGuQ8kuc9pgc9s8qqaq=dirpe0xb9q8qiLsFr0=vr0=vr0dc8meaabaqaciaacaGaaeqabaqabeGadaaakeaacqWGzbqwdaWgaaWcbaGaemyAaKMaemOAaOgabeaakiabg2da9maalaaabaGaemiwaG1aaSbaaSqaaiabdMgaPjabdQgaQbqabaaakeaacqGGOaakdaaeWbqaaiabdIfaynaaDaaaleaacqWGPbqAcqWGQbGAaeaacqaIYaGmaaaabaGaemOAaOMaeyypa0JaeGymaedabaGaemOBa4ganiabggHiLdGccqGGPaqkdaahaaWcbeqaaiabigdaXiabc+caViabikdaYaaaaaaaaa@46C2@ where *i *= 1, ..., *q *and *j *=1, ..., *n*.

Utilizing the primary Φ columns of this *Y *matrix, the data can be embedded as points in ℝ^Φ^.

### Additional DR (projection) methods

Two implementations of PCA were calculated for comparison: correlation and covariance. These calculations were conducted with the *prcomp *function in the 'stats' package of R [29]. Since classical MDS is synonymous to PCA calculated on a Euclidean distance matrix, nonmetric MDS was performed instead (based on ranking of dissimilarities), to avoid redundant information and for an additional comparison with another nonlinear approach, using the *isoMDS *[31] function in the 'MASS' package of R [29]. Kernel PCA was calculated with a Gaussian radial basis function kernel using the *k.pca *function in the 'kmethods' package of R [29]. This kernel function was chosen to maintain consistency with the kernel used in both spectral methods. The weighted graphs Laplacian for the two spectral methods are anchored on some form of a Gaussian kernel (see **Spectral methods **section for the difference between the kernels).

### Comparison of transformed distances

In order to better demonstrate how each of the three DR methods with a kernel function transforms Euclidean distances to its respective weighting values (e.g. Gaussian radial basis function, weighted graph Laplacian *L *matrix-Ng *et al*., weighted graph Laplacian *K *matrix-Lafon), an example was generated with simulated data. A data matrix of five observations was generated (each composed of 10 variables) and the three methods with a kernel function were calculated on the data matrix to compare between the final transformed weight matrix that is decomposed (i.e. *svd*) and the standard Euclidean distance matrix (Table 3). This evaluation illustrates how distances within each method are transformed in the final step prior to decomposition, and shows where on the distribution local and remote distances converge in this transformation. For example, in kernel PCA, a Gaussian radial basis function kernel is computed from the distance matrix and these entries are plotted against their respective Euclidean distances to represent the transformed space that the eigenfunctions are calculated on, in order to provide a low dimensional embedding. For both weighted graphs Laplacian from Ng *et al*. and Lafon, the weight values in the *L *and *K *matrix, respectively, are compared against the Euclidean distance matrix.

**Table 3:** Euclidean distance matrix for simulated example

	**1**	**2**	**3**	**4**	**5**
**1**	0.0	5.4	26.8	31.4	56.4
**2**	5.4	0.0	30.2	35.6	59.5
**3**	26.8	30.2	0.0	12.2	39.1
**4**	31.4	35.6	12.2	0.0	40.0
**5**	56.4	59.5	39.1	40.0	0.0

The Euclidean matrix in Table 3 contains 10 unique distance values. Two values are less than the 25% of the distribution, three values are greater than the 75% of the distribution, and the remaining values are within the interquartile range. This range of distances is utilized to convey both the subtle and apparent differences within the transformed space between methods.

**Figure 6:**
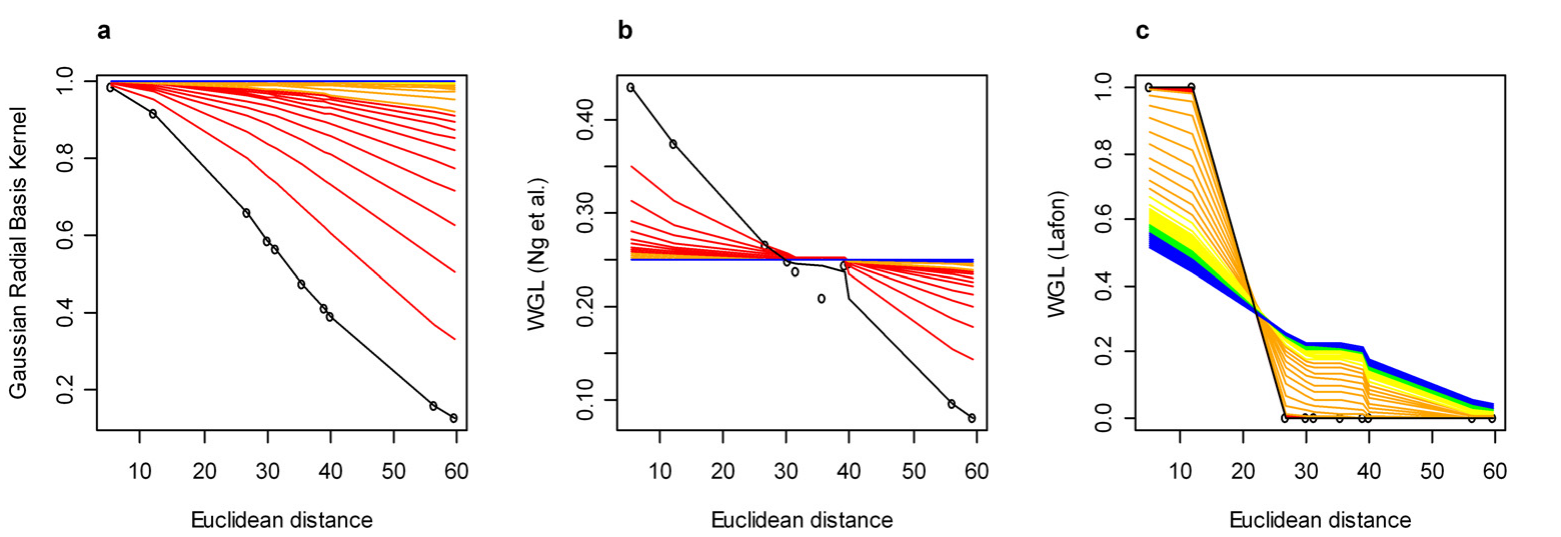
**Weighted values vs. Euclidean distance**. For each plot, the black trend line is drawn based on ordering the values for each vector (weighted values and Euclidean distance values); this gives a general fitting of each curve. The portion of the rainbow spectrum (ROYGB) shading for the lines are drawn by the same criterion, however, each line is calculated based on a set of increasing epsilon smoothing values. For example, epsilon values are increased from the 1% quantile to the 50% quantile of the squared Euclidean distance distribution, where each set of 10% values are plotted with a separate color in the rainbow spectrum, staring at red and ending at blue. This method of line shading illustrates how the transformed Euclidean distances are adjusted across a dynamic range of epsilon values for each method. **(a) **Gaussian radial basis function kernel values vs. Euclidean distances. **(b) **Entries in the matrix of the weighted graph Laplacian, *L *from Ng *et al*., vs. Euclidean distance. **(c) **Entries in the matrix of the weighted graph Laplacian, *K *from Lafon vs. Euclidean distance.

The results are shown in Figure 6. For each plot, the points represent the relationship between the transformed space (y-axis) and Euclidean distance (x-axis). The trend lines in each plot are determined by ordering both the x and y axis vectors, so an ordered transformation from Euclidean distance values to the weighted values would be depicted with a line that passes through each point. Those plots that do not exhibit this line pattern do not maintain the identical ordering from distances to weights. The latter statement does not imply that there is either a disadvantage or an advantage to the method. It simply provides a means of comparison for those methods that distort the ordering of some distances when transformed into a weight value. The black line in each plot is calculated based on assigning the epsilon smoothing term to the minimum non-zero squared Euclidean distance (*ε *= min_*i*≠*j *_||*x*_*i *_- *x*_*j*_||^2 ^> 0). Each subsequent line that is shaded by a portion of the rainbow spectrum (ROYGB) is then calculated by increasing this epsilon term by 1% (determined from the distribution of the squared Euclidean distances) increments up to the 50% of the distribution of squared Euclidean distances. Each line color is assigned to bins of 10% incremental values from 1% to 50% (e.g. weight value lines calculated with epsilon = 1%–10% values from the distribution of squared Euclidean distances are shaded red, epsilon = 11%–20% values from the distribution of squared Euclidean distances are shaded orange, etc.).

From the kernel PCA plot in Figure 6a, a Gaussian radial basis function demonstrates convergence of local Euclidean distances in the transformation to weight values, while remote distances exhibit more linearity with these weight values. In addition, as the epsilon smoothing term is increased at very small values, local distances converge to similar weight values much faster than remote distances. This example illustrates how local distance structure is better preserved at levels specific to the epsilon smoothing term utilized, than remote distances. Small distances are collapsed to a similar weighted value, while large distances maintain relatively stronger linearity with Euclidean distances, though this is reduced as the epsilon smoothing term is increased to values greater than the 10% of the distribution of squared Euclidean distances (denoted by the change in line shading from red to orange). The two smallest Euclidean distances converge to similar values rather quickly as the epsilon smoothing term is increased, while the points in the distance distribution converge more slowly. At epsilon values greater than the 20% (line shading of yellow, green, and blue) of the distribution of squared Euclidean distances, the weight values all converge to the same value of 1.

The Laplacian matrix, *L *from Ng *et al*., in Figure 6b shows that at minimal values of the epsilon smoothing term, there is near linearity between Euclidean distances and values in the matrix *L*. However, as the smoothing epsilon term is increased, the convergence occurs with both local and remote distances, while the points in the middle of this distribution maintain similar values (represented by the approximate slope of 0 from the values within the interquartile range of the plot). Both tails of the trend line quickly approach a weight value of ~0.25 with epsilon values at less than 20% of the distribution of squared Euclidean distances (denoted by line shading of only red and orange). This example illustrates that values within the middle region of the distribution (as compared the right and left tails of the distribution) are transformed to similar weight values in matrix *L*, with rather small values of the smoothing epsilon term, and then as this epsilon value is increased to only slightly larger values, all points in the function converge to a similar weight value. In addition, the Laplacian matrix, *L *does not preserve the same ordering of Euclidean distances.

The Laplacian matrix, *K *from Lafon (Figure 6c), in contrast to the matrix *L*, demonstrates convergence of weight values on both extremes of the Euclidean distance vector at very small values of the epsilon smoothing term, as illustrated by the small variance in red lines (i.e. epsilon vales at less than the 10% of the distribution of squared Euclidean distance). Additionally, opposite to the matrix *L*, these weight values become more linear (less converged) on both right and left tails of the distribution as the epsilon smoothing term is *increased *to values greater than the 20% of the distribution of squared Euclidean distances (line shading corresponding to colors of yellow, green, and blue). It is interesting to note that at maximal epsilon values (indicated by yellow, green, and blue lines in Figure 6c), the function generated by the weight values in the *K *matrix resemble the function generated by the weight values in the *L *matrix at the minimum epsilon value (indicated by the black line in Figure 6b). However, similar to the *L *matrix values, the ordering of Euclidean distances is not preserved. This example illustrates how the Lafon method differs from the other two, in that maximal convergence of both local and remote distances is optimized at minimal epsilon values (i.e. values at less than 10% of the distribution of squared Euclidean distance for this example). Based on the partitioned structure in the data, this reduces the dynamic range of Euclidean distances, particularly along the middle of the distribution, and transforms the distance structure to weight values (in matrix *K*) at both extremes of the distribution. Such a transformation, that acts to create a large gap between local and remote distances (at a partitioning threshold driven by the data) is shown to provide an optimal distance transformation for subsequent spectral decomposition for purposes of elucidating meaningful structure in image seriation and microarray species classification examples presented. Since the convergence of local and remote distances in the *K *matrix is highly dependent upon the primary partitioning point (for this example, between the Euclidean distances of 12.2 and 26.8), it is assumed that the most meaningful structures are defined by the difference between the smallest two Euclidean distances and the remaining eight Euclidean distances.

### Classifiers

The *k*-nearest neighbor (KNN) algorithm (for *k *= 2 and 3) was calculated for 1,000 independent trials for each method (to address the problem of random assignment when ties occur in nearest neighbor voting) and the mean error was computed using the *knn *function in the 'class' package of R [29]. The discriminant analysis classifiers both for separate variances (QDA) and pooled variances (LDA) were computed using the *qda *and *lda *functions, respectively, in the 'MASS' package of R [29]. All classification models were trained and tested with leave-one-out cross-validation (LOO-CV).

## Authors' contributions

BWH conducted the data analysis, created the visuals, and was involved in drafting the manuscript. Both JW and JLS were involved in the revisions, edits, and critically assessing the manuscript for technical and general content. All authors read and approved the final manuscript.
